# Prospective pilot study of Floseal® for the treatment of anterior epistaxis in patients with hereditary hemorrhagic telangiectasia (HHT)

**DOI:** 10.1186/s40463-019-0379-y

**Published:** 2019-10-15

**Authors:** John M. Lee, Vincent Wu, Marie E. Faughnan, Andrea Lasso, Andrea Figol, Shaun J. Kilty

**Affiliations:** 1 Division of Rhinology, Department of Otolaryngology – Head and Neck Surgery, St. Michael’s Hospital, University of Toronto, 190 Elizabeth Street, Rm 3S-438, TGH RFE Building, Toronto, Canada; 2 Li Ka Shing Knowledge Institute, St. Michael’s Hospital, Toronto, Canada; 3 Toronto HHT Centre, Division of Respirology, Department of Medicine, St. Michael’s Hospital, University of Toronto, Toronto, Canada; 4 Department of Otolaryngology – Head and Neck Surgery, Ottawa Hospitals, University of Ottawa, Ottawa, Canada; 5 Dr. S. Kilty Medicine Professional Corporation, Ottawa, Canada

**Keywords:** Hereditary hemorrhagic telangiectasia, HHT, Epistaxis, Floseal®

## Abstract

**Background:**

Epistaxis is the most common symptom of hereditary hemorrhagic telangiectasia (HHT), affecting more than 98% of adults with HHT, with significant impact on quality of life. Floseal® has been shown to be effective for the management of anterior epistaxis, but has yet to be thoroughly evaluated in this population. Our goal was to evaluate the efficacy of Floseal® for managing acute anterior epistaxis in patients with HHT.

**Methods:**

A pilot prospective clinical trial was conducted at two tertiary referral centres, St. Michael’s Hospital, Toronto, Canada and The Ottawa Hospital, Ottawa, Canada. All patients with HHT presenting with acute anterior epistaxis to the two study centres, who enrolled in the study, received Floseal® treatment. The primary outcome measures were achievement of hemostasis and changes in the Epistaxis Severity Score (ESS) between baseline and one-month follow up. Secondary outcome measure included clinical assessment of the nasal cavity.

**Results:**

Seven patients were included in the final analysis. All patients underwent treatment of anterior epistaxis with Floseal® and achieved control of epistaxis within 15-min post-application. Application of Floseal® was well tolerated, with patients reporting a pain score of 3 ± 3.13 out of 10. There was no statistically significant difference noted in ESS scores pre-treatment and one-month follow up, 6.27 ± 2.42 vs. 4.50 ± 2.44, *p* = 0.179. There was a significant improvement clinically on exam of the nasal cavity between baseline and at one-month follow up, indicated by a decrease in the clinical assessment score, 17.29 ± 7.70 vs. 9.57 ± 7.81 (*p* = 0.0088).

**Conclusions:**

Patients with HHT presenting with acute epistaxis were able to achieve hemostasis with one application of Floseal®, with the procedure being very well tolerated with minimal pain. Although there was no significant change in ESS scores, clinical assessment of the nasal cavity revealed significant improvement at one-month follow up post treatment with Floseal®.

**Trial registration:**

This multi-centered prospective clinical trial was registered with ClinicalTrials.gov (NCT02638012). Registered on December 22, 2015.

## Introduction

Hereditary hemorrhagic telangiectasia (HHT) is an autosomal dominant disorder that is characterized by abnormal blood vessel development. This manifests as mucocutaneous telangiectasias and visceral arteriovenous malformations (AVM). The prevalence of HHT is 1 in 5000–10,000 people, with some geographic variability [1–3]. The most common symptom that patients with HHT experience is epistaxis, eventually affecting more than 98% of adults [1, 4]. On average, HHT patients experience 18 episodes of epistaxis per month [5]. Quality of life (QoL) in patients with HHT has been found to be significantly decreased [6]. Epistaxis severity correlates directly with this decrease in QoL for patients with HHT [6, 7].

Various methods for management of chronic epistaxis in patients with HHT have been described in the literature. These include medical therapy such as hormonal manipulation, antifibrinolytic agents, and vascular endothelial growth factor inhibitors along with surgical interventions such as laser coagulation, septodermoplasty, and nasal closure with the Young’s procedure [1]. In the acute setting, the mainstay of management for active epistaxis has involved a combination of direct pressure, nasal packing, possible cauterization, and fluid resuscitation [1, 8].

Floseal® Hemostatic Matrix combines two independent hemostatic agents, and has been used extensively in nasal and skull-base surgical procedures to achieve hemostasis [9]. The gelatin granules within Floseal® provide an initial tamponade effect, while the high concentrations of human thrombin converts fibrinogen into fibrin, thereby accelerating blood clot formation [10]. With regards to the management of anterior epistaxis, Floseal® has been shown in a randomized trial to be non-inferior to standard nasal packing, which is the current standard of care [11]. In comparison to anterior nasal packing, Floseal® has been shown to be cost-effective for anterior epistaxis treatment [12]. Its utility has also been demonstrated in the management of posterior epistaxis as well [13]. However, patients with HHT were not evaluated in these trials.

Patients afflicted with HHT can suffer from frequent and heavy epistaxis, often requiring hospitalization for transfusions and surgical or endovascular procedures. As HHT patients represent a distinct group of individuals who are at increased risk of epistaxis, which has significant negative impact on QoL, evaluation within this specific group was warranted. Herein, we performed a pilot prospective clinical trial evaluating the efficacy of Floseal® in managing acute epistaxis in HHT patients.

## Methods

This multi-centered prospective clinical trial was registered with ClinicalTrials.gov (NCT02638012). Research ethics approval was received from both St. Michael’s Hospital and The Ottawa Hospital.

### Study population

We aimed to recruit 10 patients, which is the sample size that is deemed appropriate for pilot studies [14]. Patients age 18 and above with a documented diagnosis of HHT, and who were experiencing active anterior epistaxis were approached to voluntarily participate in this study. Patients were recruited after the Otolaryngology – Head and Neck Surgery service was consulted, either through the Emergency Department, or from another in-patient service. Patients were excluded if they i) had a known sensitivity to Floseal®, ii) had a known sensitivity to the topical medications administered as part of the evaluation and treatment of epistaxis (lidocaine, xylometazoline hydrochloride) or iii) were pregnant and/or breast feeding (the safety of Floseal® has not been established in pregnant women).

### Floseal® treatment

The nasal cavity was suctioned, and the bleeding was visualized with anterior rhinoscopy. A total of 5 mL of Floseal® (one standard preparation) was applied under direct visualization into the affected nasal cavity using the provided application catheter by a senior study investigator (JML or SK). Anterior nasal pressure was then applied for 5 min, and the nasal cavity was then re-inspected 15 min after the initial application. In the event that bleeding was not controlled, an additional Floseal® application was to be applied. With failure to control bleeding with 2 applications of Floseal® the protocol was to remove gel and clots with suction, and the patient was to be treated with a standard packing treatment (absorbable or non-absorbable) as standard of care.

### Outcome measures

A patient-reported epistaxis questionnaire was administered as part of the study, and included the Epistaxis Severity Score (ESS). The survey is attached as part of Additional file 1. Modification to the ESS questionnaire included changes with regards to the timing (1 month, as compared to 3 months in the original ESS questionnaire). The patient-reported questionnaire was administered to all patients at the time of the Floseal® application (baseline), and at 1 month following treatment. Patients were also asked to report pain associated with application of Floseal®, rated on a visual-analogue scale from 0 to 10, with 0 being “no pain”, and 10 being “worst pain in your life”.

The primary outcome measures were achievement of hemostasis and changes in the ESS score between baseline and one-month follow up. Secondary outcome measures assessed subjective changes in epistaxis symptoms between baseline and follow up. Additionally, patients were reassessed clinically at 1 month follow up, capturing changes in telangiectasias, crusting, scarring, and active bleeding sites in the nasal cavity. Both sides of the nose were scored separately in each of these domains from 0 to 10, with 0 being “none” and 10 being “severe”. Clinical assessments were only performed by senior study investigators (JML and SK). The clinical assessment form is included as part of Additional file 2.

### Statistical analysis

Descriptive statistics were used to summarize the frequency and percentage of categorical variables. Continuous variables were reported as mean and standard deviation. Paired t-test was performed to compare baseline and one-month follow up differences in ESS, frequency of nose bleeds, and severity of nose bleeds. All statistical analyses were performed using Prism (v.7, GraphPad, USA), with significance set to α = 0.05.

## Results

A total of 8 patients were initially approached and recruited for the study. Six of the 8 patients had unilateral anterior epistaxis, while two patient had bilateral anterior epistaxis. All patients underwent treatment of anterior epistaxis with the application of Floseal®. There was control of epistaxis, with hemostasis achieved at 15-min post-application of Floseal® in all patients. Only one application of Floseal® was required in all cases. One patient with bilateral anterior epistaxis was unable to complete the patient-based outcome measures and clinical follow-up. Therefore, only 7 patients were included for data analysis.

Mean age was 61.6 ± 12.0, with 5 males (71.4%). All patients had anemia and chronic gastrointestinal bleeding secondary to their diagnosis of HHT. Three patients had pulmonary AVM, and one patient had hepatic AVM. Other baseline comorbidities included hypertension in three patients, asthma in two patients, and a diagnosis of cancer in one patient. None of the patients were anticoagulated at the time of presentation for epistasis.

The severity of the acute bleed was assessed to be 5.13 ± 3.00, out of a possible maximum of 10. Patients tolerated the application of Floseal® well, reporting a pain score of 3.00 ± 3.13, out of a possible maximum of 10. There were no adverse events encountered with the application of Floseal®.

In comparing normalized ESS scores, there was no statistically significant difference noted between pre-treatment and one-month follow up scores, 6.27 ± 2.42 vs. 4.50 ± 2.44, *p* = 0.179. Subjectively, two patients reported improved epistaxis symptoms, while 5 reported similar epistaxis symptoms after Floseal® treatment. During the follow up period, all patients had additional epistaxis episodes, that were stopped by self-administered methods. These included anterior nasal pressure, petroleum ointment, nasal packing, tissue paper, or a combination of the aforementioned. Two of the patients continued taking tranexamic acid orally at time of follow-up.

With clinical assessment of telangiectasias, crusting, scarring, and active bleeding sites in the nasal cavity, there was a significant decrease in severity between pre-treatment levels and at one-month follow up, 17.29 ± 7.70 vs. 9.57 ± 7.81 (*p* = 0.0088). None of the patients had active bleeding noted at time of follow up.

## Discussion

This was the first study to prospectively assess the use of Floseal® for the management of acute anterior epistaxis in patients with HHT. Epistaxis is the most common symptom with an associated significant negative impact on QoL in this patient population. Traditionally, nasal packing has been utilized for the management of epistaxis in HHT patients. However, packing is an undesirable method of treatment in this patient cohort, as it is associated with increased trauma to the friable nasal mucosa, higher rates of secondary re-bleeding with packing removal, and significant pain and discomfort [11, 13, 15–17].

Warner et al. (2014) reported on the domiciliary use of Floseal® for self-treating acute anterior epistaxis in the home setting for patients with HHT. The authors found that its use was well tolerated and preferred by patients, and its use was able to prevent hospital admissions [14]. However, Floseal® in this case was self-applied, outside of a controlled study setting. In our study, we were able to confirm the efficacy of Floseal® in achieving hemostasis for acute anterior epistaxis in our series under controlled settings. Our series highlights that if Floseal is applied to the active bleeding site, it provides a high success rate for treatment of acute anterior epistaxis in patients with HHT. Furthermore, the application of Floseal® in our series was well tolerated, with a low reported pain score and no reported adverse events. An evaluation of the cost-effectiveness of Floseal® by Le et al. found that compared to standard nasal packing, within the Canadian publically funded healthcare system, the use of Floseal® was considered overall more cost-effective despite the increased per unit cost [12]. The authors concluded this was directly related to the greater number of recurrent epistaxis episodes averted [12]. Although the economic evaluation did not focus on HHT patients, we can extrapolate the given the increased frequency and recurrent nature of epistaxis in HHT patients, the cost-effectiveness of Floseal may also apply to this population. Overall, the findings reiterate previously described advantages of Floseal® as a less painful alternative to traditional packing for the management of epistaxis and more cost-effective, thereby potentially representing a paradigm shift away from nasal packing as the treatment of choice in HHT patients [11, 13, 15, 17].

When evaluating the potential effect of being treated with Floseal® on the ESS score (which combines the frequency, duration, severity, and sequelae from epistaxis) we did not find a statistically significant difference between the pre-treatment ESS score and follow up ESS score at one-month post-treatment. This finding aligns with the primary role of Floseal®, which acts as a tissue matrix to tamponade bleeding and achieve rapid hemostasis. As noted, hemostasis was achieved in all patients in our study with one application. Floseal® offered control of epistaxis in the acute setting. However, not surprisingly, it did not appreciably change chronic symptoms in HHT.

With regards to clinical assessment of the anterior nasal cavity, there was a significant improvement in the appearance of the nasal cavity post-treatment with respect to telangiectasias, crusting, scarring, and active bleeding sites in the nasal cavity. Importantly, we noted that none of the patients had active bleeding at the time of their follow-up. When active bleeding was excluded from the analysis, with clinical assessment focused only on telangiectasias, crusting and scarring, there was no statistically significant difference between the pre-treatment and post-treatment scores (*p* = 0.359). Bleeding, then, was seen as a cofounding variable between the baseline and follow-up clinical assessment scores. Nevertheless, results from the clinical assessment again reiterate that the usefulness of Floseal® as a treatment is restricted to the acute hemorrhagic episode and does not alter the chronicity of the disease for patients with HHT.

With regards to the study participants, there was a patient who was initially recruited into the study after presenting to the Emergency Department with significant bilateral anterior epistaxis. Floseal® was applied and hemostatis was achieved. However, this patient also had significant bleeding from the oral cavity, necessitating additional urgent interventions including vessel embolization and critical care admission. The patient was not included in the final study analysis, as we were unable to capture patient-reported outcomes, given the acute deterioration in the patient’s overall health status secondary to other sources of bleeding. However, it is important to note that again that during the episode of acute epistaxis, one application of Floseal® was sufficient in achieving hemostasis.

There are several potential limitations to this study. Firstly, given the small sample size and study design, it may be difficult to generalize the study results to the HHT population for the management of acute anterior epistaxis. We encountered difficulty recruiting patients during the two-year study period given the HHT disease prevalence, stringent treatment protocol, and the tendency for patients with HHT to self-manage their frequent anterior epistaxis episodes on their own, out of hospital. In light of the results, which showed that FloSeal® was able to stop acute bleeds in 100% of HHT patients presenting to the two study centers, we believed that additional recruitment may have delayed the dissemination of our study results to providers who care for patients with HHT and seeking novel management for acute epistaxis. Moreover, blinding could not be performed in this study given the nature of Floseal® application. As a result, there is the possibility of reporting bias from both patients and the study investigators. Furthermore, this study did not assess the long term patient-based or clinician-based outcomes associated with FloSeal® use, as it was mainly directed towards assessing acute epistaxis management. Studies in the future can aim to compare the use of Floseal® to other treatment methods for acute anterior epistaxis management for patients with HHT, improve on the overall sample size, and possible introduction of blinding of clinical assessors.

## Conclusion

Floseal® application achieved hemostasis, after a single application, in this small series of HHT patients presenting with acute anterior epistaxis. Furthermore, Floseal® application was well tolerated with minimal discomfort and without any adverse events. We conclude that Floseal® should be considered as a treatment option for the management of acute anterior epistaxis in patients with HHT.

## Supplementary information

**Additional file 1:** Epistaxis Severity Score Questionnaire.

**Additional file 2:** Clinical Assessment Form.

## Data Availability

The datasets used and/or analyzed during the current study are available from the corresponding author on reasonable request.

## Abbreviations

AVM: Arteriovenous malformation
ESS: Epistaxis Severity Score
HHT: Hereditary hemorrhagic telangiectasia
QoL: Quality of life
