# Supplementary material for: Prospective pilot study of Floseal® for the treatment of anterior epistaxis in patients with hereditary hemorrhagic telangiectasia (HHT)
Source: J Otolaryngol Head Neck Surg. 2019 Oct 15;48:48. doi: 10.1186/s40463-019-0379-y (PMC6794791; doi:10.1186/s40463-019-0379-y)
Supplement: Supplementary file 1 — Additional file 1: Epistaxis Severity Score Questionnaire. [file 40463_2019_379_MOESM1_ESM.docx]

**Additional file 1: Epistaxis Severity Score Questionnaire**

1. How often did you typically have nosebleeds during the past one month?

- Less than once per month
- Once per month
- Once per week
- Once per day
- Several per day

1. How long did each nosebleed typically last for you during the past one month?

- < 1 minute
- 1-5 minutes
- 6-15 minutes
- 16-30 minutes
- >30 minutes

1. How would you describe your typical nosebleed intensity during the past one month?

- Not typically gushing or pouring
- Gushing or pouring

1. Have you sought medical attention outside of this research study for your nosebleeds during the past one month?

- No
- Yes

1. Are you anemic (low blood count) currently?

- No
- Yes

1. Have you received a red blood cell transfusion specifically for nosebleeds during the past month?

- No
- Yes
