# Supplementary material for: Prospective pilot study of Floseal® for the treatment of anterior epistaxis in patients with hereditary hemorrhagic telangiectasia (HHT)
Source: J Otolaryngol Head Neck Surg. 2019 Oct 15;48:48. doi: 10.1186/s40463-019-0379-y (PMC6794791; doi:10.1186/s40463-019-0379-y)
Supplement: Supplementary file 2 — Additional file 2: Clinical Assessment Form. [file 40463_2019_379_MOESM2_ESM.docx]

**Additional file 2: Clinical Assessment Form**

**Telangiectasias:**

Right None 0—1—2—3—4—5—6—7—8—9—10 Severe

Left None 0—1—2—3—4—5—6—7—8—9—10 Severe

**Crusting:**

Right None 0—1—2—3—4—5—6—7—8—9—10 Severe

Left None 0—1—2—3—4—5—6—7—8—9—10 Severe

**Synechiae:**

Right None 0—1—2—3—4—5—6—7—8—9—10 Severe

Left None 0—1—2—3—4—5—6—7—8—9—10 Severe

**Active Bleeding Sites:**

Right None 0—1—2—3—4—5—6—7—8—9—10 Severe

Left None 0—1—2—3—4—5—6—7—8—9—10 Severe
